# Prion protein fragment (106–126) activates NLRP3 inflammasome and promotes platelet-monocyte/neutrophil interactions, potentially contributing to an inflammatory state

**DOI:** 10.3389/fcell.2025.1534235

**Published:** 2025-02-25

**Authors:** Rashmi Verma, Jyotsna Kailashiya, Avijit Mukherjee, Rameshwar Nath Chaurasia, Debabrata Dash

**Affiliations:** ^1^ Centre for Advanced Research on Platelet Signaling and Thrombosis Biology, Department of Biochemistry, Institute of Medical Sciences, Banaras Hindu University, Varanasi, India; ^2^ Department of Neurology, Institute of Medical Sciences, Banaras Hindu University, Varanasi, India

**Keywords:** prion, platelets, NLRP3 inflammasome, platelet-monocyte/neutrophil interaction, ROS, EGCG, caspase-1

## Abstract

**Introduction:**

Prion diseases are neurodegenerative disorders where infectious prion proteins (PrP) featuring an amyloidogenic amino acid sequence, PrP (106–126), accumulate in the brain leading to neuroinflammation while it can also access circulation by breaching the blood-brain barrier. Platelets are highly sensitive cells in blood, which have been widely employed as “peripheral” model for neurons. In addition to their stellar roles in hemostasis and thrombosis, platelets are also known to function as immune cells and possess necessary components of functional inflammasome. This study demonstrates that prion proteins drive inflammasome assembly in platelets and upregulate activity of caspase-1, a critical readout of functional inflammasomes.

**Methods:**

Flow cytometric analysis was performed to measure intracellular ROS levels, caspase-1 activity, and platelet-monocyte/neutrophil interactions. Microscopy was used to assess the co-localization of NLRP3 and ASC.

**Results:**

Inflammasome activation is fuelled by reactive oxygen species (ROS) generated in prion-stimulated platelets that eventually leads to formation of platelet-monocyte/neutrophil aggregates, which was prohibited by small-molecule inhibitors of either caspase-1 or ROS.

**Discussion:**

Thus, in addition to their neurotoxic effects on neuronal cells and stimulation of pro-coagulant activity in platelets, prions also unleash an inflammatory response in the organism.

## Introduction

Prion disease is a neurodegenerative disorder caused by the scrapie form of prions (PrP^sc^), which induces abnormal folding of cellular prion (PrP^c^) leading to protease-resistant β-pleated sheets (Sandberg et al., 2011). In cerebrospinal fluid concentration of prion protein ranges from 1.5 ng/mL to 546 ng/mL (Vallabh et al., 2019) while prions in circulation are likely sourced from platelets (Robertson et al., 2006), endothelial cells (Simák et al., 2002) and lymphocytes (Prinz et al., 2003), which carry membrane-bound PrP^c^ released into the bloodstream. PrP can cross the blood-brain barrier in both directions, i.e., either from blood-to-brain or brain-to-blood (Banks et al., 2009). The synthetic PrP (106–126) resembles PrP^sc^ in several ways as it carries a protease-resistant amyloidogenic β-pleated sheet. Thus, PrP (106–126) has been extensively employed as a model for PrP^sc^-related research (Ettaiche et al., 2000; Gautam et al., 2022; Gu et al., 2002; Mallick et al., 2015; Melo et al., 2007).

Human platelets are anucleate blood cells essential for hemostasis; however, hyperactivity of platelets leads to pathological thrombus formation. Besides hemostatic activities, platelets also play a seminal role in innate and adaptive immune responses (Semple et al., 2011). Activated platelets interact with circulating neutrophils and monocytes that are critical mediators of inflammation and innate immunity, thereby driving an inflammatory phenotype. Platelets express numerous pattern recognition receptors, including toll-like receptors (TLRs) and nucleotide-binding oligomerization domain-like receptors (NLRs), which enable them to act as circulating cellular sensors that provide a unique link between hemostasis and inflammation (Zhang et al., 2015). NLRs recognize pathogen/damage-associated molecular patterns (PAMPs/DAMPs) and homeostasis-altering molecular patterns (HAMPs). Inflammation is directly linked with inflammasome assembly that produces pro-inflammatory cytokines, interleukin (IL)-1β and IL-18. NLRP3 inflammasome is a multimeric protein complex consisting of NLRs, apoptosis-associated speck-like protein (ASC), and pro-caspase-1. Inflammasome assembly instigates caspase-1 activation, which results in proteolytic cleavage of pro-IL-1β and pro-IL-18 into their active mature forms, IL-1β and IL-18, respectively.

Platelets possess all the components required for assembly of an inflammasome complex (Qiao et al., 2018). Of these, NLRP3 in platelets has received the greatest attention and has been linked to a variety of illnesses, such as sepsis (Cornelius et al., 2020), dengue (Hottz et al., 2013) and sickle cell disease (Vogel et al., 2018). Oxidative stress plays a significant role in promoting the activation of inflammasome complexes (Zhao and Zhao, 2020). Calcium overload has been reported to impair mitochondrial function and generate excessive ROS in sepsis resulting in inflammasome activation (Zhao et al., 2020). Epigallocatechin-3-Gallate (EGCG), a polyphenol and green tea extract known for its antioxidant properties, also inhibits prion-mediated neurotoxicity (Lee et al., 2015) and has recently been reported as an inhibitor for NLRP3 inflammasome (Zhang et al., 2021)**.** NLPR3 also contributes to platelet activation, aggregation, and thrombus formation *in vitro* (Murthy et al., 2017) and regulate integrin outside-in signaling (Qiao et al., 2018).

We have previously reported that prion proteins unleash a prothrombotic state in platelets associated with raised intracellular calcium and microvesicle release (Mallick et al., 2015), which is restrained in the presence of fibrinogen (Gautam et al., 2022). It was reported that prion protein (100 µM) also activates NLRP3 inflammasome in microglial cells (Shi et al., 2012), which is linked to its neurotoxicity (Hafner-Bratkovič et al., 2012). Additionally, as platelets can be exposed to PrP^C^ released from their α-granules upon stimulation (Starke et al., 2005), we queried whether prion might also play a role in activating the platelet inflammasome. In this study, we report that exposure to PrP fuels inflammasome assembly and caspase-1 activation in platelets mediated through an upsurge in ROS, that leads to formation of platelet-monocyte/neutrophil aggregates unleashing an inflammatory phenotype.

## Materials

Prion peptide (106–126) (KTNMKHAGAAAAGAVVGGLG) was purchased from Biomatik, United States. Antibodies against NLRP3 (#NBP2-12446), and ASC (#NBP1-78977APC) were from Novus Biologicals, United States. Antibodies against IL-1β (#12242) and β-actin (#A2066) were procured from Cell Signaling Technology and Sigma, respectively. HRP-conjugated goat anti-rabbit and anti-mouse IgG were the products of Bangalore Genei. APC-Mouse anti-human CD41a (#559777), FITC-Mouse anti-human CD14 (#555397), FACS lysis solution and FACS Flow sheath fluid were from BD Biosciences. Calcein AM (#C3100MP) and goat anti-rabbit IgG (Alexa Flour 488-conjugated) (#A11008) were procured from Invitrogen. Permanox slides (#160005) were from ThermoFisher. The FAM-FLICA Caspase-1 Assay kit (#SKU 97) was from ImmunoChemistry Technologies. Fibrinogen (Fg, #4883), YVAD-CHO (#400011), thrombin receptor-activating peptide (TRAP, #S1820), 2′,7′-dichlorodihydrofluoresceindiacetate (H_2_DCFDA, #D6883), epigallocatechin gallate (EGCG, #E4143), N-acetyl L-cysteine (NAC, #A7250), IgG (#5006), ethylenediaminetetraacetic acid (EDTA), sodium orthovanadate, dimethylsulfoxide (DMSO), Triton X-100, prostaglandin E1 (PGE1), paraformaldehyde (PFA) were purchased from Sigma while bovine serum albumin (BSA) were from HIMedia. All reagents were of analytical grade. Type I deionized water (18.2 MΩ cm, Millipore) was used for preparation of solutions. Experiments were carried out strictly as per the guidelines of Institutional Ethical Committee.

## Methods

### Platelet preparation

Peripheral venous blood was collected from healthy volunteers (both male and female participants with age varying between 20 and 40 years) in acid citrate dextrose (trisodium citrate, 74.8 mM; citric acid, 38.06 mM; and dextrose, 136 mM). Platelets were isolated by differential centrifugation. Blood was centrifuged at 100 g for 20 min. PGE1 (1 µM) and EDTA (2 mM) were added to the supernatant (platelet-rich plasma, PRP). PRP was centrifuged at 800 g for 7 min. Cells were washed in buffer A (20 mM HEPES, 134 mM NaCl, 2.9 mM KCl, 1 mM MgCl_2_, 5 mM glucose, 0.34 mM NaH_2_PO_4_, 12 mM NaHCO_3_, 0.35% BSA, 1uM PGE1, pH 6.2) and centrifuged again at 800 g for 7 min. Finally, cells were suspended in buffer B (pH 7.4), which was the same as Buffer A but without PGE1 and BSA. Cell count was maintained at 2–4 × 10^8^ cells/mL by an automated cell counter (Multisizer 4, Beckman Coulter). Leucocyte contamination was found to be less than 0.015%. All steps were executed under sterile conditions, and precautions were taken to maintain the cells in a resting state.

### Preparation of prion (106–126) fragment

PrP (106–126) (KTNMKHMAGAAAAGAVVGGLG) was dissolved in 1 mL buffer B (please see above) at 0.5 mM stock concentration and stored at −20°C in aliquots. In the untreated control counterparts (resting platelets), buffer B substituted prion solution as the vehicle.

### Assessment of Caspase-1 activity

The activity of caspase-1 was determined using a fluorescent inhibitor of caspase-1 (FLICA), whose structure is represented by 5-carboxyfluorescein-Tyr-Val-Ala-Asp-fluoromethylketone (FAM-YVAD-FMK). FLICA is cell-permeant dye that covalently interacts with ‘active’ caspase-1 through YVAD sequence emitting green fluorescent signal while inhibiting further enzymatic activity. Unbound dye diffuses out of the cell during the wash steps. Thus, the degree of fluorescence reflects the extent of caspase-1activity. Staining was carried out as per the manufacturer’s protocol. In brief, prior to exposure to PrP (50 µM for 20 min at RT) platelets were incubated with either EGCG (5 µM) or NAC (1 mM) for 10 min at 37°C, followed by incubation with FLICA reagent for 30 min at 37°C in the dark. Stained cells were washed, followed by fixation. Cells were suspended in sheath fluid and analyzed on a flow cytometer (FACSCalibur, BD Biosciences).

### Measurement of intracellular ROS

Platelets were incubated with H_2_DCFDA for 30 min at RT in the dark. H_2_DCFDA enters the cell, is deacetylated by esterases, and oxidized by ROS to highly fluorescent 2′,7′- dichlorofluorescein (DCF). Platelets were pre-treated with either EGCG (5 µM) or NAC (1 mM) for 10 min at 37°C, followed by incubation with either PrP (50 µM) or buffer B (vehicle) for 20 min at RT. Population of stained cells were analyzed by flow cytometry (FACSCalibur, BD Biosciences). Data were evaluated using CellQuest Pro Software as described earlier (Chaurasia et al., 2022).

### Co-localization of ASC and NLRP3

Platelets pre-treated with either prion (50 µM) or buffer B (vehicle) were allowed to adhere to Permanox slides for 30 min, washed thrice with 1X PBS, and fixed with 4% PFA for 10 min. Following three washes, platelets were permeabilized with 0.1% Triton X-100 for 1 min. Slides were blocked with 2% BSA for 1 h, washed and incubated with anti-NLRP3 antibody (1:50) overnight at 4°C, washed thrice, and then exposed to anti-rabbit Alexa Flour 488-conjugated secondary antibody (1:100) for 2 h at RT. Slides were rewashed and incubated with APC-conjugated rabbit anti-ASC antibody for 2 h at RT. Controls were processed identically except for the omission of primary antibodies. Slides were examined under Zeiss LSM 700 laser scanning confocal microscope with ×63 oil objective (numerical aperture, 1.40) and 1 AU pinhole size. Results were representative of three different experiments.

### Analysis of platelet-monocyte/neutrophil interaction

Platelet-monocyte/neutrophil (PMN) interaction was evaluated as described earlier (Ekhlak et al., 2023). Fresh human blood (20 μL) was added to a cocktail containing 10 μL each from APC-anti-CD41a (platelet-specific) and FITC-anti-CD14 (leucocyte-specific) antibodies and mixed gently. Then it was treated with either EGCG (5 µM) or YVAD (1 µM), followed by incubation with TRAP (2 μM) or PrP (50 µM) for 15–20 min at RT. RBCs were lysed with 800 μL FACS lysis solution (1X, BD Biosciences) for 10 min at RT. PMN interaction was analysed on a flow cytometer. Side scatter voltage was set at 350 with a threshold of 52 V. A dot plot of side scatter (SSC) versus log FITC-CD14 fluorescence was created using the CellQuest Pro software. Amorphous gates were drawn for monocyte (high fluorescence and low SSC) and neutrophil (low fluorescence and high SSC) populations. Fluorescence data were acquired from each sample using four-quadrant logarithmic amplification for 1,000 events in either neutrophil or monocyte gate and analysed with CellQuest Pro Software.

## Results

### Prion stimulates assembly of NLRP3 inflammasome complex and caspase-1 activation in human platelets

**FIGURE 1:**
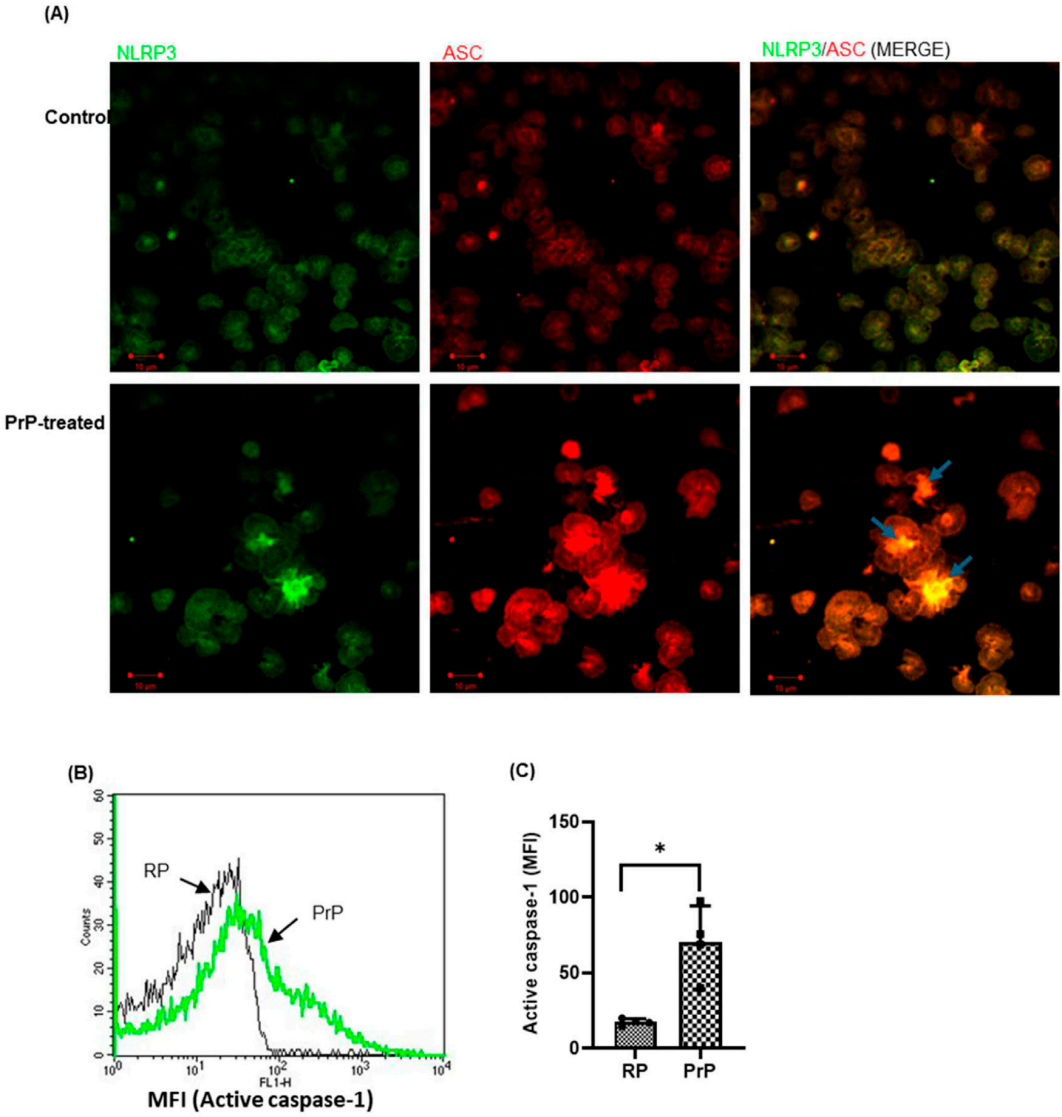
Prion promotes inflammasome assembly in platelets. **(A)** Confocal images representing inflammasome assembly in platelets treated either with prion or buffer B (Control). NLRP3 (green) and ASC (red) proteins were dispersed uniformly in control platelets while PrP treatment allowed focal clustering of these proteins prompting brighter visualization (scale bar, 10 µm). Arrows indicate co-localization of NLRP3 and ASC in PrP-treated platelets. RP, resting platelets. Images are representative of three independent experiments. **(B, C)** PrP (50 µM) provoked robust caspase-1 activation. The bar graph represents mean ± SEM (n = 4). *P < 0.05 as compared to RP, analysed by paired Student’s t-test.

We have earlier demonstrated that, prion induces platelets to adopt a procoagulant state (Gautam et al., 2022; Mallick et al., 2015). In the present study, we further investigated the role of prions in transforming these cells to an inflammatory phenotype. Exposure of platelets to PrP (106–126) (50 µM) for 20 min brought about focal accumulations of NLRP3 and ASC, the key components of the inflammasome, in platelet cytosol (Figure 1A), reflective of the complex formation (Vogel et al., 2018)). This was associated with significant enhancement in caspase-1 activity (by 296%) in PrP-treated platelets (Figures 1B, C). Our previous study demonstrates that prion promotes platelet agglutination rather than aggregation (Mallick et al., 2015), this might be the reason behind the clustering of cells in PrP-treated platelets (Figure 1A).

### Inflammasome activation in prion-stimulated platelets is fuelled by the generation of reactive oxygen species

**FIGURE 2:**
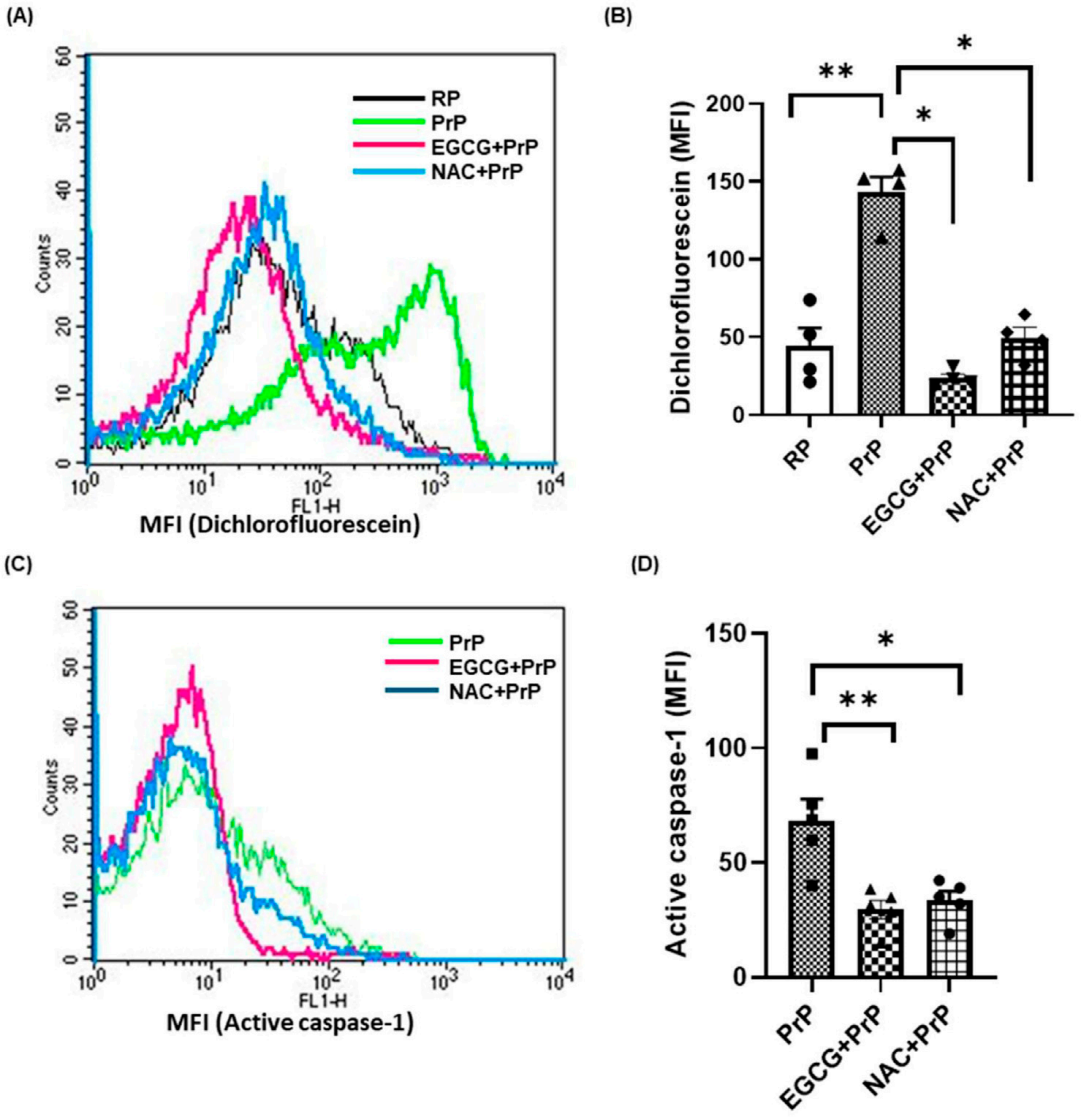
ROS plays a key role in inflammasome activation in platelets. **(A, B)** PrP (106–126) (50 µM) induced a significant rise in intracellular ROS in platelets, which was prevented upon pre-treatment with either EGCG (5 µM) or NAC (1 mM). **(C, D)** PrP-induced caspase-1 activity in platelets was significantly prohibited by either EGCG or NAC. RP, resting platelets treated with buffer B. Data are presented as mean ± SEM and are representatives of at least four different experiments. Statistical analysis was performed using repeated measures one-way ANOVA (paired), with Sidak’s multiple comparison test. *P < 0.05; **P < 0.01.

We next investigated the underpinning molecular mechanism governing inflammasome activation in prion-challenged platelets. ROS is known to play a vital role in inflammasome activation (Abais et al., 2015). Level of platelet intracellular ROS was found to be augmented by 200% upon exposure to PrP (106–126). The rise was significantly blunted by 84% and 65%, respectively, upon pre-treatment of cells with either EGCG (5 µM) or NAC (1 mM), the known scavengers of ROS (Figures 2A, B). Next, we interrogated the role of ROS in driving prion-mediated inflammasome signaling. Prior exposure to either EGCG or NAC prohibited caspase-1 activity by 57% and 51%, respectively, thus implicating prion-ROS-inflammasome-caspase-1 signaling axis in transformation of resting platelets to functionally active inflammatory units (Figures 2C, D). It is noteworthy to state here that, PrP (106–126) induces ROS generation and caspase-1 activation in a dose-dependent manner evaluated at 20 μM and 50 µM prion concentrations (Supplementary Figure 3).

As prion induces NLRP3 inflammasome activation (signal 2) in platelets, it prompted us to ask whether it also instigates the priming signal (signal 1) that involves synthesis of pro-IL-1β and NLRP3. Although LPS has been shown to promote synthesis of pro-IL-1β in anucleate platelets (Brown et al., 2013; Lindemann et al., 2001), the necessity of signal-1 for inflammasome initiation in platelets has been a subject of debate (Hottz et al., 2013). We did not observe synthesis of either pro-IL-1β or NLRP3 in response to PrP (106–126) in platelets (Supplementary Figure 2), thus ruling out the induction of priming signal in these cells.

### Inflammasome activity in prion-treated platelets drives platelet-monocyte/neutrophil interaction

**FIGURE 3:**
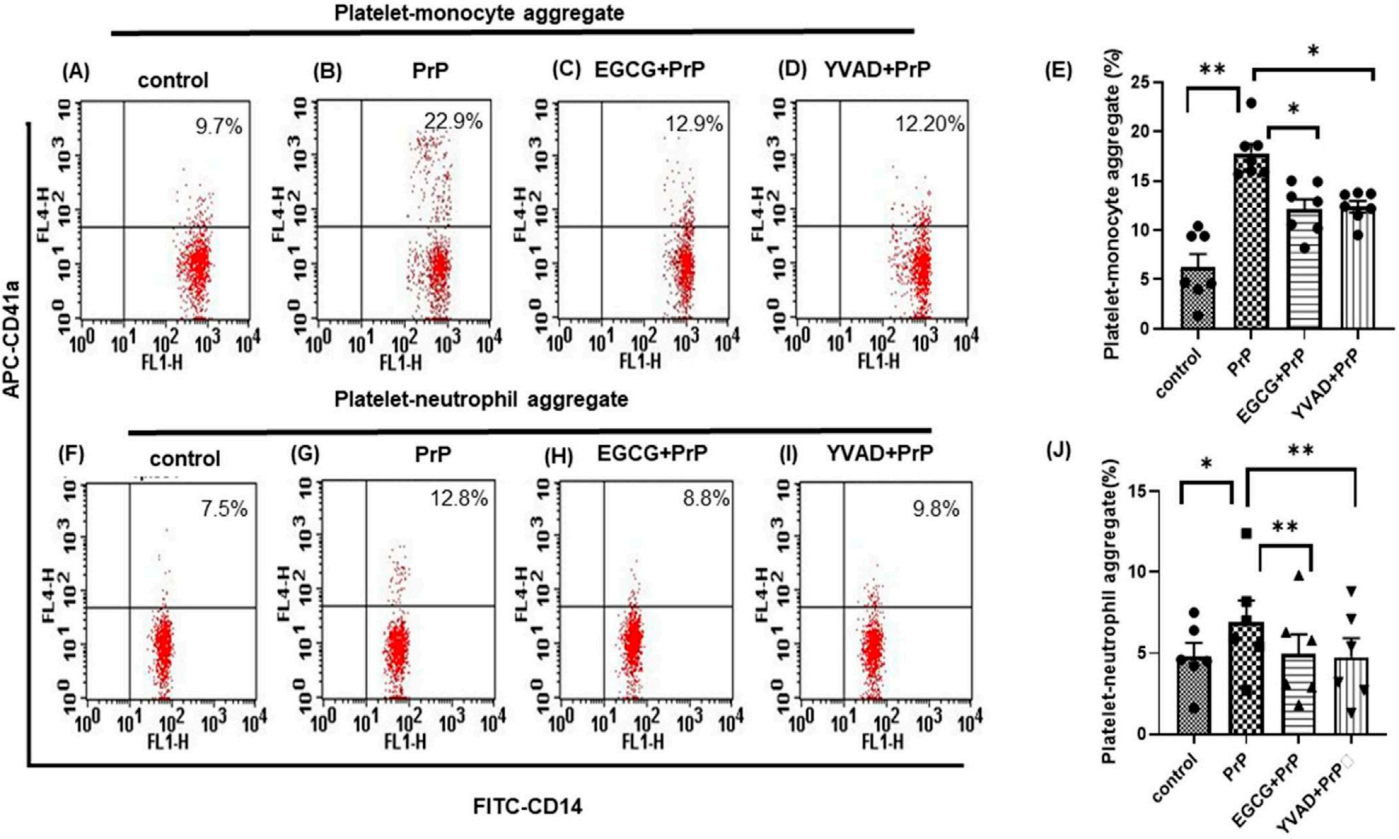
Prion prompts platelet-monocyte/neutrophil (PMN) aggregate formation. Flow cytometric analysis of platelet-monocyte **(A–D)** and platelet-neutrophil **(F–I)** interactions in whole blood stained with anti-CD41a-APC (specific for platelets) and anti-CD14-FITC (specific for neutrophils/monocytes), followed by treatment with PrP (106–126) (50 µM) in presence or absence of either EGCG (5 µM) or YVAD (1 µM), as indicated. Control platelets were treated with buffer B (vehicle) in place of PrP. Amorphous gates were drawn for monocyte (high fluorescence and low SSC) and neutrophil (low fluorescence and high SSC) populations. **(E)** (n = 7) and **(J)** (n = 6), bar diagrams showing the percentage of platelet-monocyte and platelet-neutrophil aggregate formation, respectively (mean ± SEM). *P < 0.05, **P < 0.01, as analysed by repeated measures one-way ANOVA (paired) with Dunnet’s multiple comparison test.

Platelets interaction with circulating monocytes and neutrophils is a sensitive index of the state of platelet activity (Cerletti et al., 2012; Ortiz-Muñoz et al., 2014). PMN interaction generates a pro-inflammatory phenotype by activating leucocytes and the emergence of neutrophil extracellular traps (NETs) (Margraf and Zarbock, 2019). Circulating amyloid-β, another β pleat-rich peptide, reportedly activates platelets leading to a localized inflammatory response by promoting NET formation (Canobbio et al., 2017). Since prion peptide upregulated NLRP3 inflammasome activity in platelets, we asked whether it would provoke interaction with leucocytes. Predictably, platelet agonist TRAP (2 µM) prompted a surge in platelet-monocyte as well as platelet-neutrophil aggregate formation, which was impaired by YVAD, a specific inhibitor of caspase-1 (Supplementary Figure 1), thus linking inflammasome to platelet-monocyte/neutrophil (PMN) interaction. Prion peptide, too, propelled platelet-monocyte and platelet-neutrophil interactions significantly by 178% and 39%, respectively (Figure 3). These prion-instigated events were forestalled either by the ROS-scavenger EGCG (by 31% and 29%, respectively), or by YVAD (by 33% and 32%, respectively) (Figure 3), thus implicating the prion-ROS-caspase-1 axis in evolution pro-inflammatory phenotype.

**FIGURE 4:**
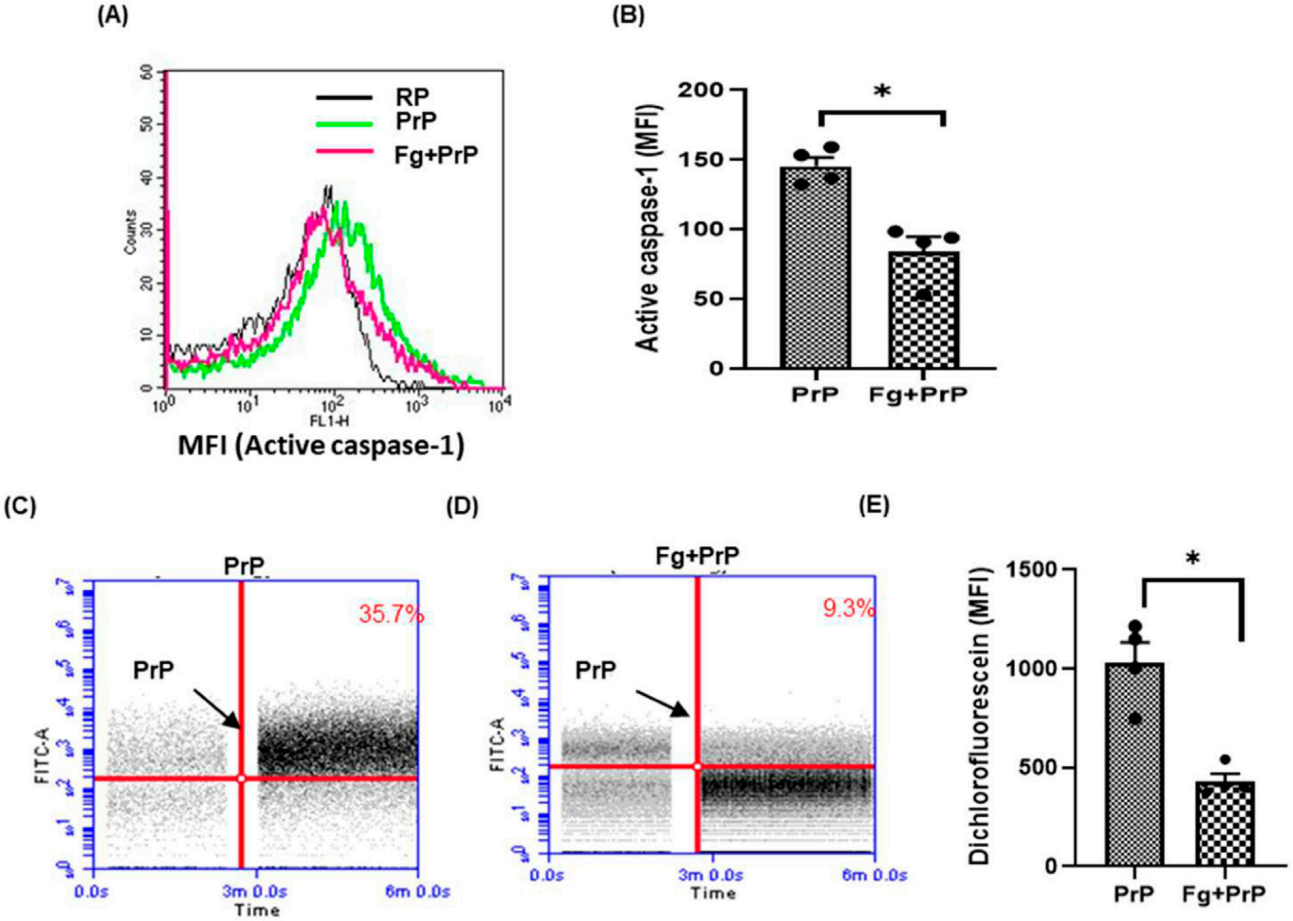
Fibrinogen prohibits prion-induced caspase-1 activation and generation of ROS. **(A, B)** prion-induced caspase-1 activity in the absence or presence of fibrinogen (Fg) (2 mg/mL). Fibrinogen was incubated for 10 min before adding PrP (106–126) (50 µM). **(C–E)** prion-induced surge in Intracellular ROS in platelet population in the absence or presence of fibrinogen, respectively. The bar graphs represent mean ± SEM from four independent experiments. RP, resting platelets treated with buffer B. *P < 0.05 as compared with PrP-treated platelets, analysis by paired Student’s t-test.

Several laboratories including ours have demonstrated high-affinity interaction between fibrinogen and β pleat-rich peptides such as prion and amyloid-β that prohibits action of these proteins on neuronal cells, as well as on platelets (Ahn et al., 2014; Ahn et al., 2010; Gautam et al., 2022; Sonkar et al., 2014). Keeping with above, we observed a 42% decline in prion-induced caspase-1 activity when platelets were pre-treated with fibrinogen (Figures 4A, B), reflective of impaired inflammasome activation. Concordantly, prion-mediated ROS generation, too, was restrained by 58% upon prior exposure of platelets to fibrinogen (Figures 4C–E). However, fibrinogen was unable to restrict prion-mediated PMN interaction (data not shown), which could be attributable to factors in blood interacting with either fibrinogen or PrP, thus precluding their association.

## Discussion

Platelets express approximately 2000 PrP^c^ on their surface, while they also store them in their α-granules (Starke et al., 2005). Platelet activation triggers the release of stored PrP^c^ from granules, augmenting prion surface count to as many as 4,500 molecules per platelet and raising potential for its interaction with nearby platelets (Holada et al., 1998). Prions can also cross the blood-brain barrier and enter vasculature, exposing themselves to blood cells in circulation (Banks et al., 2009). Diseases associated with protein misfolding such as prion disease, Alzheimer’s disease, type-2 diabetes, and Parkinson’s disease are marked by inflammatory responses (Shi et al., 2015). Misfolded proteins trigger cellular stress (Rao and Bredesen, 2004), which promotes inflammasome activation (Shi et al., 2015). Prion has also been shown to promote the transcription of DAMP receptors in neuronal cells (Carroll et al., 2018) and activate NLRP3 inflammasome in microglial cells (Shi et al., 2012). The effect of prion on platelets is not a novel concept as we have earlier identified its role in hemostasis (Gautam et al., 2022; Mallick et al., 2015; Shi et al., 2012). Beyond their primary contribution to hemostasis, platelets are also recognized as immune effector cells, which induce NLRP3 inflammasome activation in innate immune cells like monocytes (Rolfes et al., 2020). Previous research has demonstrated that inflammasome complex formation in activated platelets facilitates the progression of dengue and sickle cell disease (Hottz et al., 2013; Vogel et al., 2018). These findings inspired us to explore the role of misfolded prion protein in inflammasome signaling in platelets.

Oxidative stress plays a crucial role in the conversion of PrP^c^ to PrP^sc^ (Prasad and Bondy, 2019). Prion promotes ROS generation and Ca^2+^ metabolism in neuronal cells (De Mario et al., 2019) while increased calcium level provokes higher production of ROS (Görlach et al., 2015). In the CNS, oxidative stress and neuroinflammation are two of the main pathological hallmarks associated with multiple neurodegenerative diseases (Braidy et al., 2022). We have already demonstrated that prion instigates an increase in the level of intracellular free calcium in platelets (Mallick et al., 2015), which prompted us to examine the cytosolic oxidant status in these cells. In present study, prion was found to facilitate intracellular ROS production in platelets, which was inhibited in the presence of either of two ROS scavengers, EGCG and NAC. EGCG, known for its anti-oxidant property (Mokra et al., 2022), also inhibits NLRP3 inflammasome (Zhang et al., 2021). It has anti-prion activity (Barreca et al., 2018) and protects against prion protein-induced damage by regulating autophagy (Lee et al., 2015). We found that prion promotes inflammasome complex formation by generating active caspase-1, which subsequently triggers a pro-inflammatory response in platelets. ROS scavengers (NAC and EGCG) effectively attenuate caspase-1 activation, which underscores their potential in addressing inflammatory disorders. Inflammasome assembly was validated in our study from co-localization of NLRP3 and ASC proteins, and cleavage of pro-caspase-1 into its enzymatically active form.

In a recent study, blocking inflammasome activation was reported to attenuate proliferation and invasion of circulating pro-inflammatory monocytes in cases of stroke (Baek, 2024). PrP^sc^ activates microglia and astrocytes to release pro-inflammatory cytokines, namely, IL-1β, IL-8, TNF-α, and IL-6, unleashing an inflammatory phenotype. Activated platelets engage with leucocytes through numerous surface receptors that include P-selectin on platelets ligating with P-selectin glycoprotein ligand-1 (PSGL-1) expressed on leucocyte membrane. Similarly, CD40L, GPIIb/IIIa and GPIb, respectively, allow platelets and platelet-derived extracellular vesicles (PEVs) to interact with CD40, ICAM-1 and Mac-1 receptors present on leucocytes. In recent years, PEVs have emerged as a significant source of non-coding regulatory RNAs in the blood (Tao et al., 2017), which are capable of binding to leucocytes (Fendl et al., 2018). We have earlier demonstrated significant shedding of PEVs from prion-challenged platelets (Mallick et al., 2015). Here we demonstrate that prion promotes platelet-monocyte/neutrophil interaction, which is repressed either by EGCG or YVAD, the inhibitor of caspase-1.

Pro-thrombotic attributes of platelet-monocyte/neutrophil interaction have already been recognized (Ghasemzadeh and Hosseini, 2013). Thrombus is a dynamic structure composed of platelets, RBCs, leucocytes, fibrin, and von Willebrand Factor, with platelets and fibrin playing seminal roles in its formation and stability. However, the sizeable presence of prions within the thrombus milieu sourced from platelets cannot be disregarded. Research into this area could lead to newer insights into role of prions linked to inflammation and thrombogenesis. In conclusion, we found that prion stimulates ROS production in platelets leading to activation of caspase-1 and platelet-monocyte/neutrophil aggregate formation, thus implicating a prion-ROS-inflammasome-caspase-1 signaling axis in transformation of resting platelets to functionally active inflammatory units. However, considerable caution must be used in drawing such a conclusion due to limitations like only one type of prion fragment being employed in the study and experiments being performed only *ex vivo* on isolated platelets and whole blood.

## Data Availability

The original contributions presented in the study are included in the article/Supplementary Material, further inquiries can be directed to the corresponding author.
