# Supplementary material for: Prion protein fragment (106–126) activates NLRP3 inflammasome and promotes platelet-monocyte/neutrophil interactions, potentially contributing to an inflammatory state
Source: Front Cell Dev Biol. 2025 Feb 25;13:1534235. doi: 10.3389/fcell.2025.1534235 (PMC11895701; doi:10.3389/fcell.2025.1534235)
Supplement: Supplementary file 1 [file DataSheet1.docx]

**Supplementary Material**

**Prion protein fragment (106–126) activates NLRP3 inflammasome and promotes platelet-monocyte/neutrophil interaction, potentially contributing to an inflammatory state**

Rashmi Verma^1^, Jyotsna Kailashiya^1^, Avijit Mukherjee^1^, Rameshwar Nath Chaurasia^2^ and Debabrata Dash^1*^

^1^Center for Advanced Research on Platelet Signaling and Thrombosis Biology, Department of Biochemistry, Institute of Medical Sciences, Banaras Hindu University, Varanasi-221005, India

^2^ Department of Neurology, Institute of Medical Sciences, Banaras Hindu University, Varanasi-221005, India

^*^ Correspondence: Prof. Debabrata Dash, Center for Advanced Research on Platelet Signaling and Thrombosis Biology, Department of Biochemistry, Institute of Medical Sciences, Banaras Hindu University, Varanasi-221005, Uttar Pradesh, India. Email: (ddash.biochem@gmail.com)

**Supplementary Information**

**Supplementary Methods**

**Western Blotting**

Platelets were lysed in Lammeli lysis buffer followed by boiling. Briefly explained, platelet proteins were separated on 12% SDS PAGE and electrophoretically transferred onto the PVDF membrane. After blocking, membranes were incubated with primary antibodies (anti-IL-1β, 1:1000; anti-NLRP3, 1:1000 and anti-β-actin, 1:5000) overnight at 4°C on the shaker. After washing, membranes were incubated with specific HRP-conjugated secondary antibodies. Antibody binding was detected by using enhanced chemiluminescence. Images were acquired on a multispectral imaging system (Biospectrum 800 imaging system, UVP) and quantified using Vision Works LS software (UVP).

**Cell viability assay**

A fluorescent dye, Calcein AM, detected cell viability assay. Calcein AM is a hydrophobic compound capable of crossing the cell membrane of living cells. Inside the cell, it is hydrolyzed by esterase enzymes into calcein, a fluorescent molecule that remains trapped within the cell. In contrast, dead cells either do not produce calcein or produce it at much lower levels. After Prion treatment, platelets were incubated with calcein AM (3 µg/ml) for 15 min in the dark at RT. Samples were analyzed using the flow cytometer (Becton Dickinson, model Accuri C6 + ).

.


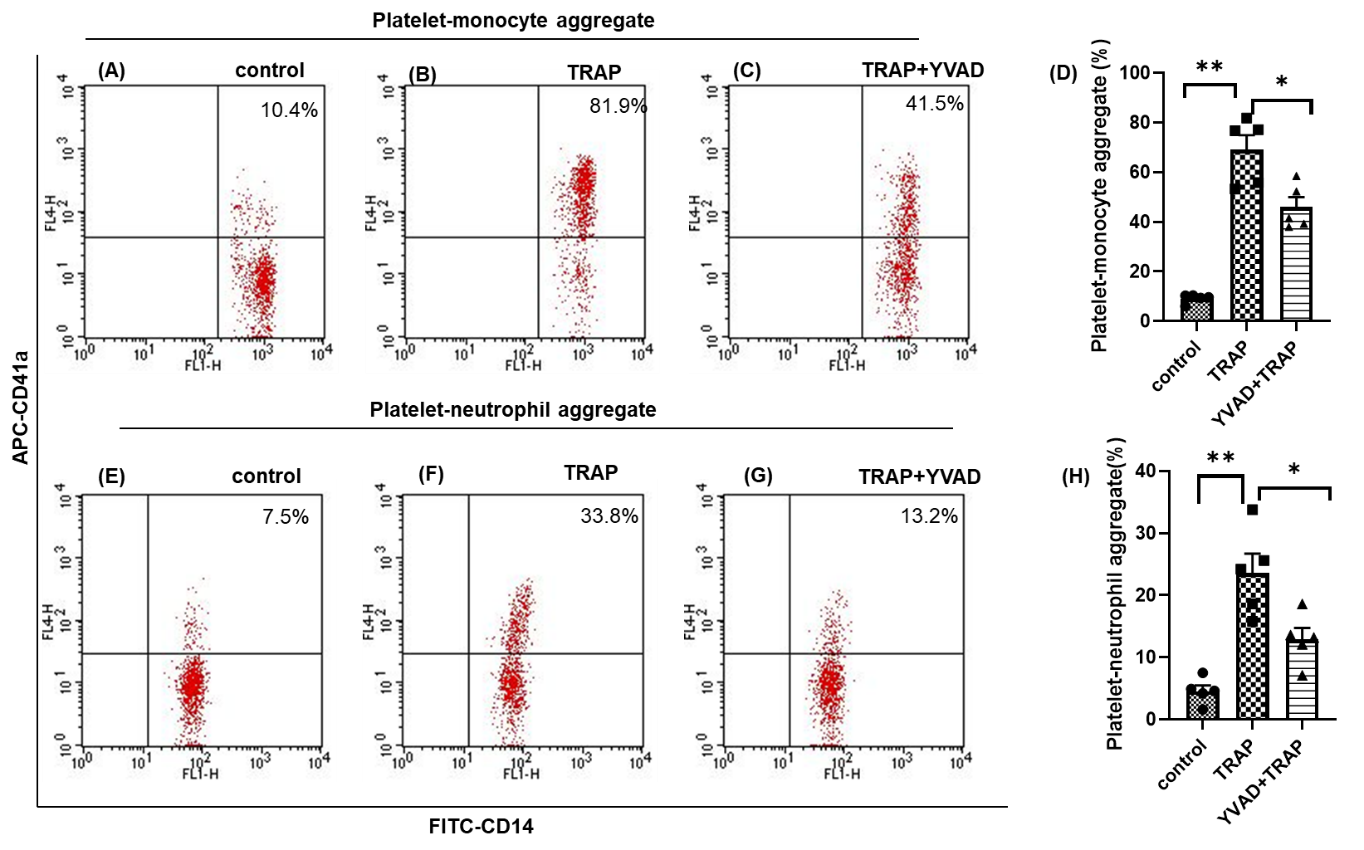


**Supplementary Fig.1**. **TRAP-induced platelet-leucocyte interaction is regulated by caspase-1.** Flow cytometric analysis of platelet-monocyte (A-C) and platelet-neutrophil (E-G) interactions in whole blood as described in the legend to Fig. 3. TRAP (2 µM, 15 min) significantly boosted platelet-monocyte and neutrophil aggregate formation, which were significantly attenuated upon pre-treatment with YVAD (1 µM, 10 min). D and H, corresponding bar graphs presented as mean + SEM. Statistical analysis was performed using repeated measures of one-way ANOVA (paired) with Sidak’s multiple comparison test, where *P<*0.05 was considered statistically significant. **P*<0.05; ***P*<0.01.


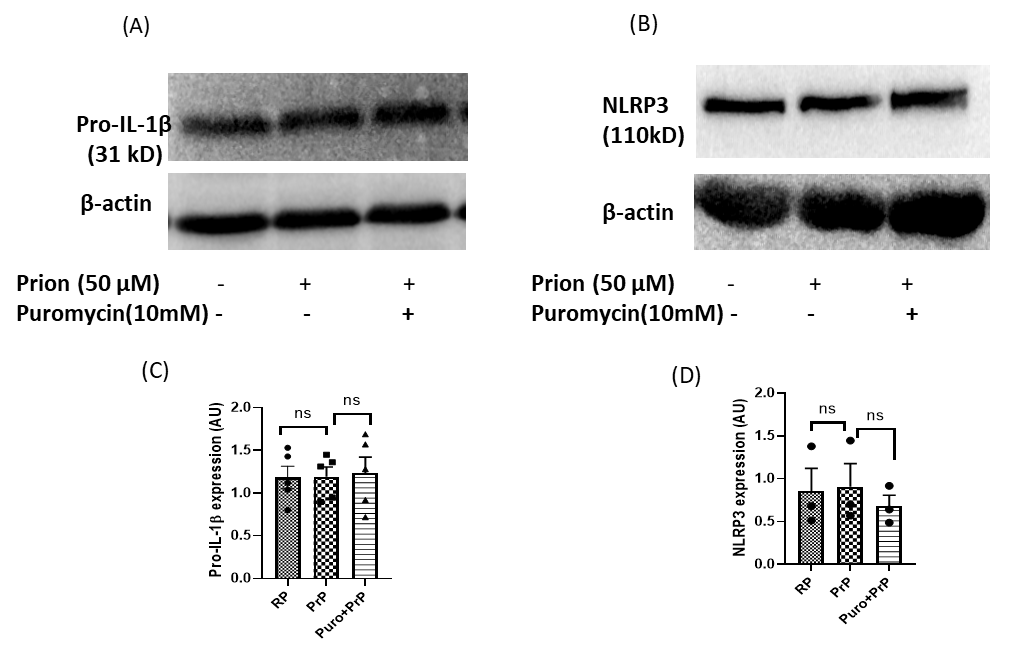


**Supplementary Fig.2. PrP (106-126)-treated platelets do not induce signal-1 activation**. A, B, Western images showing pro-IL-1β (A) and NLRP3 (B). Prion (50 µM) neither promoted the synthesis of pro-IL-1β nor NLRP3. Puromycin was employed as a negative control as it inhibits protein synthesis. Platelets were pre-incubated with puromycin for 30 min at 37◦ C before treating with prion. RP, resting platelets. Statistical analysis was performed using repeated measures of one-way ANOVA (paired) with Sidak’s multiple comparison test, where *P<*0.05 was considered statistically significant, ns stands for not significant.

**
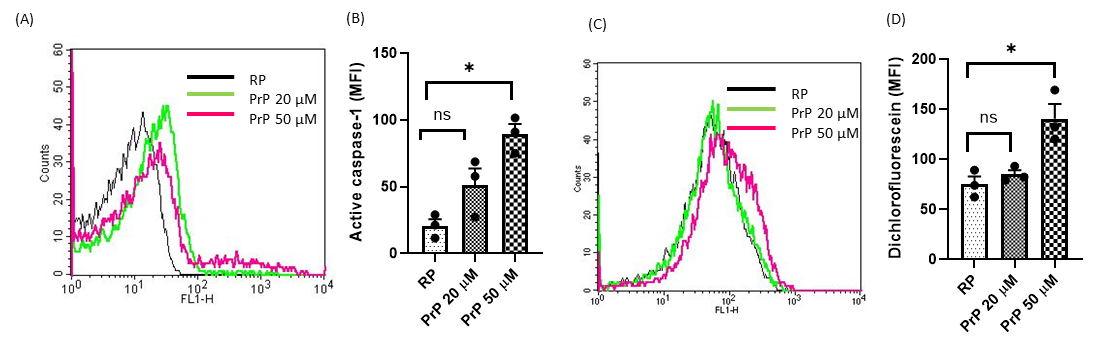
**

**Supplementary Fig.3. PrP (106-126) induces ROS generation and caspase-1 activity in platelets in a dose-dependent manner.** A, C An increase in prion concentration shows a rise in caspase-1 activity and ROS generation. B, D Bar graph represents + SEM (n=3). RP, resting platelets. Statistical analysis was performed using repeated measures of one-way ANOVA (paired) with Sidak’s multiple comparison test, where **P<*0.05 was considered statistically significant, ns stands for not significant.


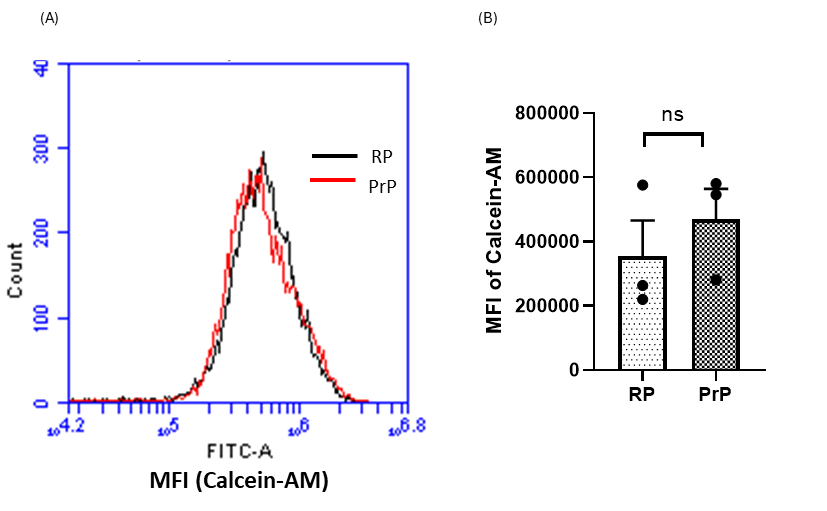


**Supplementary Fig. 4. Prion treatment does not promote cell death**. PrP (106-126) (50 µM) does not induce cellular toxicity. Prion-treated platelets exhibited similar fluorescence as compared to untreated platelets (RP, resting platelets). The bar graph represents + n=3, statistical analysis was measured by Student's *t*-test (paired), where P<0.05 was considered as statistically significant, and ns stands for not significant.


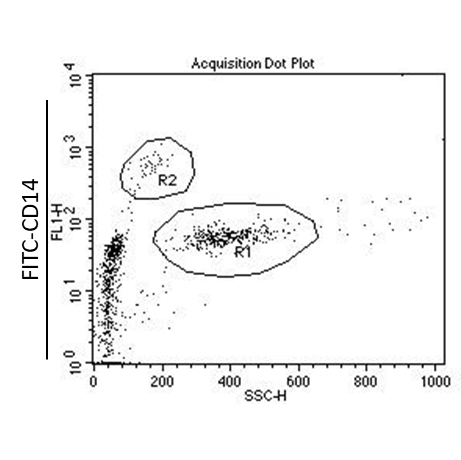


**Supplementary Fig.5. Monocyte/Neutrophil gating for study of platelet-monocyte/neutrophil interaction** FITC-labeled CD14-positive cells depict monocytes and neutrophils. In the dot plot, gate R1 denotes CD14-positive neutrophils while R2 denotes CD14-positive monocytes. CD14/CD41a dual-positive cells represent platelet monocyte/neutrophil aggregates.
